# Troglitazone suppresses telomerase activity independently of PPARγ in estrogen-receptor negative breast cancer cells

**DOI:** 10.1186/1471-2407-10-390

**Published:** 2010-07-22

**Authors:** Fariborz Rashid-Kolvear, Michael AS Taboski, Johnny Nguyen, Dong-Yu Wang, Lea A Harrington, Susan J Done

**Affiliations:** 1 Department of Laboratory Medicine and Pathobiology, Faculty of Medicine, University of Toronto, Toronto, Ontario, Canada; 2 Division of Applied Molecular Oncology, Ontario Cancer Institute/Princess Margaret Hospital, University Health Network, Toronto, Ontario, M5G 2M9, Canada; 3 Department of Medical Biophysics, Faculty of Medicine, University of Toronto, Toronto, Ontario, Canada; 4 Campbell Family Institute for Breast Cancer Research, Toronto, Ontario, M5G 2C1, Canada; 5 Department of Biochemistry and Molecular Biology, Dalhousie University, Tupper Medical Building, 5850 College St, 11-N2, Halifax, NS, B3H 1X5, Canada; 6 Wellcome Trust Centre for Cell Biology, Michael Swann Building, rm. 5.18, University of Edinburgh, Mayfield Road, Edinburgh, EH9 3JR. United Kingdom

## Abstract

**Background:**

Breast cancer is one the highest causes of female cancer death worldwide. Many standard chemotherapeutic agents currently used to treat breast cancer are relatively non-specific and act on all rapidly dividing cells. In recent years, more specific targeted therapies have been introduced. It is known that telomerase is active in over 90% of breast cancer tumors but inactive in adjacent normal tissues. The prevalence of active telomerase in breast cancer patients makes telomerase an attractive therapeutic target. Recent evidence suggests that telomerase activity can be suppressed by peroxisome proliferator activated receptor gamma (PPARγ). However, its effect on telomerase regulation in breast cancer has not been investigated.

**Methods:**

In this study, we investigated the effect of the PPARγ ligand, troglitazone, on telomerase activity in the MDA-MB-231 breast cancer cell line. Real time RT-PCR and telomerase activity assays were used to evaluate the effect of troglitazone. MDA-MB-231 cells had PPARγ expression silenced using shRNA interference.

**Results:**

We demonstrated that troglitazone reduced the mRNA expression of hTERT and telomerase activity in the MDA-MB-231 breast cancer cell line. Troglitazone reduced telomerase activity even in the absence of PPARγ. In agreement with this result, we found no correlation between PPARγ and hTERT mRNA transcript levels in breast cancer patients. Statistical significance was determined using Pearson correlation and the paired Student's *t *test.

**Conclusions:**

To our knowledge, this is the first time that the effect of troglitazone on telomerase activity in breast cancer cells has been investigated. Our data suggest that troglitazone may be used as an anti-telomerase agent; however, the mechanism underlying this inhibitory effect remains to be determined.

## Background

Excluding non-melanoma skin cancers, breast cancer is the most common malignancy in North American women. In Canada, it is estimated that there will be 22 700 new cases of breast cancer and more than 5 400 women will die from this disease in 2009 [1].

Human breast carcinomas represent a heterogeneous group of tumors with diverse behavior and responses to therapy. Many standard chemotherapeutic agents currently used to treat breast cancer are relatively non-specific and act on all rapidly dividing cells. With the recognition of different molecular subtypes of breast cancer have come efforts to develop and introduce more specifically targeted therapies such as Trastuzumab (Herceptin) in HER2-positive breast cancers. Targeted therapy has been used successfully for many years in the treatment of breast cancer. Determination of estrogen receptor (ER) status has been found to be an important predictive and prognostic factor in the management of breast cancer [2]. ER-positive breast cancer patients have a number of available anti-estrogen treatment options including tamoxifen and aromatase inhibitors. However, few effective cancer prevention and treatment strategies are available for ER-negative breast carcinoma despite the urgent need to control their more aggressive behavior. This has motivated considerable efforts toward finding new therapeutic approaches for the treatment of this group of breast cancers.

Immortalization is a necessary step toward the malignant transformation of normal human somatic cells, which have intrinsic mechanisms that monitor cell divisions and limit their life span. The terminal DNA at chromosome ends, known as telomeres, progressively shorten with each cell division and limit the replicative life span of human cells in culture [3]. Most human cancer cells maintain their telomeres through activation of telomerase (reviewed in [4]). In over 85% of human tumors, and more than 90% of breast carcinomas, telomerase is active whereas in normal tissues telomerase is active at low levels or is undetectable [5-7]. Telomerase is a large ribonucleoprotein enzyme complex with an estimated mass of approximately 670 kDa [8]. *In vitro*, two components are minimally required for human telomerase activity; telomerase reverse transcriptase (hTERT), the protein catalytic and often rate-limiting subunit of telomerase, and the telomerase RNA (hTR), an RNA template required for the synthesis of de novo telomeric DNA [9]. The inhibition of telomerase limits the growth of human cancer cells (reviewed in [4]), and various anti-telomerase strategies are currently under investigation in cancer patients.

Peroxisome proliferator activated receptors (PPARs) are members of the nuclear hormone receptor super-family, regulating gene expression via their ligand-activated transcriptional activity. There are three known subtypes of PPARs; PPARα [10], PPARβ/δ [11], and PPARγ [12]. PPARγ plays an important role in lipid metabolism, insulin sensitization, and cancers [13-15]. In addition to controlling the expression of many genes involved in lipid metabolism, and insulin sensitization, it has been found that PPARγ functions as a tumor suppressor in a variety of malignancies such as breast cancer, colon cancer, liposarcoma, ovarian cancer, and prostate cancer (reviewed in [16]).

There are two classes of ligands for PPARγ; natural ligands and synthetic ligands. The natural ligands include fatty acids and eicosanoids, components of oxidized low-density lipoproteins, oxidized alkyl phospholipids including lysophosphatidic acid and nitrolinoleic acid (reviewed in [16]). The second group are members of the thiazolidinedione (TZD) family including troglitazone, rosiglitazone, pioglitazone, and ciglitazone (reviewed in [17]). TZDs are known as insulin sensitizers and are used in the treatment of type II diabetes. It has been shown that TZDs promote the differentiation of various cell lines [18-22]. The TZDs troglitazone and ciglitazone demonstrate antiproliferative activities in several cancer models including breast cancer [23].

It has been suggested that PPARγ is a tumor suppressor gene in a variety of malignancies including breast cancer (reviewed in [16]). Based on these observations, a phase II clinical trial was undertaken using troglitazone in a group of patients with breast cancer [24]. The results showed that PPARγ activation had little clinical benefit in the women selected for the trial. However, tumour levels of PPARγ were not measured. Also, the patients had advanced breast cancer refractory to standard hormonal or chemotherapeutic agents, which may have undermined the therapeutic effect of troglitazone. The authors suggested that PPARγ might have greater impact as a preventative agent than a therapeutic agent, as in experimental studies of colon cancer.

PPARγ regulates gene expression by forming a heterodimer with retinoid X receptor (RXR) and binding to peroxisome proliferator response elements (PPRE) on target genes. Using the RXR ligand, Choi *et al. *demonstrated inhibition of cell growth and telomerase activity of breast cancer cells *in vitro *[25]. Interestingly, the PPARγ/RXR heterodimers can be activated by ligands for either PPARγ or RXR [26]. There is evidence that PPARγ can inhibit telomerase activity in some primary cells [27-29]. The role of PPARγ in modulating telomerase activity in breast cancer cells has not been studied and may have therapeutic potential. To determine if PPARγ regulates telomerase activity in breast cancer, we examined the effect of the PPARγ ligand, troglitazone, on telomerase activity in breast cancer cell lines. We showed that troglitazone reduced the mRNA expression of hTERT and telomerase activity in the MDA-MB-231 breast cancer cell line. However, we found no correlation between PPARγ and hTERT mRNA transcript levels in breast cancer patients indicating this reduction was independent from PPARγ.

## Methods

### Materials

MDA-MB-231, MCF-7, and T47D cell lines were obtained from American Type Culture Collection (ATCC) (Manassas, VA). Troglitazone and GW9662 were purchased from Sigma (Sigma-Aldrich, St Louis, MO) and BADGE from Cayman (Cayman, Ann Arbor, MI). Antibodies against Maspin and PPARγ were purchased from BD Biosciences (Mississauga, ON) and Santa Cruz Biotechnology (Santa Cruz, CA) respectively. TaqMan specific primers for PPARγ, hTERT, K19, Muc-1 were obtained from Applied Biosystems (Branchburg, New Jersey). Cell culture media was from the Ontario Cancer Institute (OCI) (Princess Margaret Hospital, Toronto, ON).

### Cell culture

MDA-MB 231 cells were cultured in alpha MEM medium (αMEM) (Princess Margaret Hospital, ON) supplemented with 10% v/v fetal bovine serum (HyClone, Logan, UT) at 37°C in a humidified atmosphere with 5% CO_2 _for 24 hours, then treated with either troglitazone (dissolved in DMSO) or an equal volume of DMSO (Sigma-Alderich Life Science, Saint Louis, MO) and incubated for various timepoints.

### Cell toxicity and cell viability assay

Cell toxicity was measured using the CellTiter96 nonradioactive proliferation assay kit (Promega, Madison). Briefly, cells were seeded in 96-well plates at a density of 7 × 10^3 ^cells/well and treated with the indicated concentrations of troglitazone. At the end of each time point, cells were incubated with 20 μl MTS/PMS solution for a further 3 hours in a humidified environment. The toxicity of troglitazone was determined by measuring the formazan produced by proliferating cells at 490 nm on a Tecan SpectraFluor Plus Plate Reader (MTX Lab Systems, Inc, Vienna, VA).

The cell viability was measured by automated Vi-CELL, which uses the trypan blue dye exclusion method (Beckman Coulter, Brussels, Belgium).

### Western blot analyzing

Total protein was extracted from cells using CytoBuster™ Protein Extraction Reagent (Novagen, Darmstadt, Germany) containing protease phosphatase inhibitors. Protein separation was performed on 4% to 12% w/v SDS-NuPage gradient gel (Invitrogen Life Technologies). Western blot analysis was performed using standard methods. Images were analyzed and quantified using Image J software [Research Services Branch (RSB) of the National Institute of Mental Health (NIMH), National Institutes of Health (NIH), USA, http://rsb.info.nih.gov/nih-image].

### Real-time RT-PCR

Total RNA was extracted using RNeasy^® ^plus kit (Qiagen, Mississauga, ON), and used for real-time reverse transcription (RT) in a two-step procedure. In the first step, an aliquot of 2 μg total RNA from each sample was reverse transcribed to cDNA using TaqMan^® ^Reverse Transcription Reagents (Applied Biosystems, Branchburg, New Jersey). In the second step, 100 ng cDNA was used for PCR using TaqMan^® ^Universal PCR Master Mix (Applied Biosystems, Branchburg, New Jersey) in a 384-well plate according to the manufacturer's instructions. We used TaqMan specific primers for hTERT, PPARγ, and GAPDH in our experiments purchased from Applied Biosystems (Warrington, UK). The real-time quantitative PCR and analysis were carried out using the ABI Prism 7900 HT Sequence Detection system (Foster City, CA).

### Stable shRNA mediated repression of PPARγ in MDA-MB-231 cells

Human *PPARγ *expression in wild type MDA-MB-231 breast cancer cells was silenced by shRNA interference. Four lentiviral gene transfer vectors expressing shRNA against *PPARγ *(NM_138712) were purchased from Open Biosystems (Huntsville, AL, USA); shPPARγ-70, shPPARγ-72, shPPARγ-73, and shPPARγ-74. An adopted Qiagen non-silencing control shRNA sequence (TTCTCCGAACGTGTCACGT) that was not complementary to any human gene was used as a control shRNA (generous gift from Dr. M.S. Tsao, University of Toronto). Lentiviruses were prepared in 293 cells followed by infection into MDA-MB-231 cells. Cells were selected using 0.5 μg/ml puromycin as previously described [30,31] and subjected to real-time RT-PCR to test the mRNA expression level of PPARγ in the infected MDA-MB-231 cells.

### Telomeric repeat amplification protocol (TRAP) assay

For TRAP assays, cells were lysed in 4-5 volumes of ice-cold CHAPS buffer (0.5% w/v CHAPS, 10 mM Tris-Cl pH 7.5, 10% v/v glycerol, 1 mM MgCl2, 5 mM b-mercaptoethanol, EDTA-free Complete protease inhibitor (Roche, Switzerland) and 400 U Roche RNase Inhibitor (Roche). TRAP assay reactions were performed using the radioactive method of the TRAPeze kit (Millipore, Billerica, MA, USA) with some modifications which follow. The telomerase extension step was performed in the absence of Taq Polymerase in the reactions. After the telomerase extension, the reactions were heated to 94°C for 2 minutes, followed by the addition of 2 units of Taq polymerase (NEB, Ipswich, MA, USA) and PCR amplification. PCR conditions were 25 cycles of 94°C for 30 seconds, 50°C for 30 seconds, and 72°C for 90 seconds. Half of the 50 μl reaction was loaded on a 10% w/v non-denaturing acrylamide: bis-acrylamide (19:1) gel in 0.6X TBE buffer. The gel was dried and exposed to a phosphorimager screen and scanned using a Typhoon Trio Imager (GE Healthcare, United Kingdom). Protein titrations of 500 ng and 250 ng were performed in each sample to ensure TRAP reaction products exhibited a semi-linear response. CHAPS lysis buffer and a positive telomerase activity control HeLa cell lysate (equivalent of 500 cells) were used as negative and positive controls respectively. The TRAP assay includes a 36 bp PCR amplification product that serves as an internal control that monitors PCR inhibition in each reaction.

### Microarray dataset and analysis

The Netherlands Cancer Institute (NKI) dataset was used to compare the expression of PPARγ and hTERT [32]. The NKI dataset has published genome-wide gene expression microarray data from 295 breast cancer samples collected between 1984 and 1995. The paired expression data of PPARγ (Probe ID: 17022) and hTERT (Probe ID: 1809) for 294 patients were analyzed by Pearson correlation. For one patient, PPARγ data was not available.

### Statistical analysis

All numerical data were expressed as median values ± SD. Statistical significance was determined by performing Pearson correlation or the paired Student's *t *test.

## Results

### Evaluating the expression of PPARγ in different breast cancer cell lines

**Figure 1:**
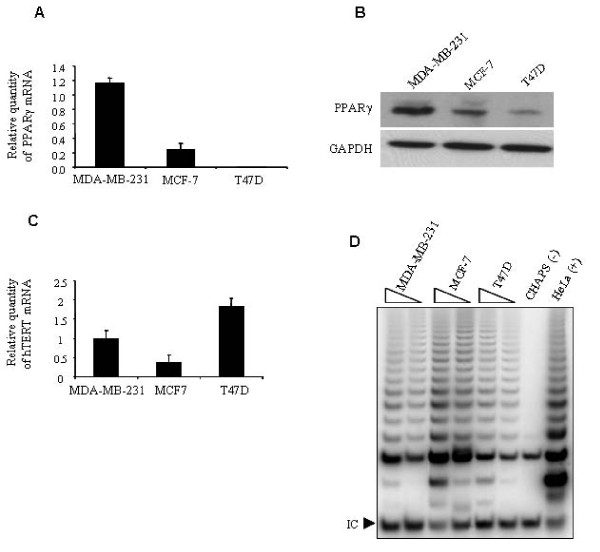
**The expression of PPARγ, hTERT and telomerase activity in several breast cancer cell lines**. **A) **Total RNA was prepared from MDA-MB-231, MCF-7, and T47D cells. Using real-time RT-PCR, the mRNA expression of PPARγ was measured and MDA-MB-231 cells had the highest expression of PPARγ mRNA. **B) **Protein lysates were prepared from the breast cancer cell lines and analyzed via western blotting to examine the level of PPARγ protein. MDA-MB-231 cells express more PPARγ protein than MCF-7 and T47D cells in this representative western blot. **C) **Total RNA was used to examine the mRNA expression level of hTERT in MDA-MB-231, MCF-7, and T47D cells by real-time RT-PCR. Values express the relative quantity of the genes to the level of mRNA expression of GAPDH. **D) **Telomerase activity was measured from protein lysates (500 and 250 ng respectively) in MDA-MB-231, MCF-7, and T47D cell lines. I.C. the internal PCR amplification control. CHAPS, lysis buffer only as a negative control. HeLa, 500 cell equivalent lysate as a positive control.

Three different breast cancer cell lines; MDA-MB-231, MCF-7, and T47D were analyzed for PPARγ expression. Real-time RT-PCR showed that mRNA expression of PPARγ was higher in MDA-MB-231 cells compared to the other two cell lines (Figure 1A). In agreement with this result, we found that the expression of PPARγ protein was higher in MDA-MB-231 than the two other cell lines (Figure 1B).

### Determining the mRNA expression level of hTERT and telomerase activity in MDA-MB-231 cells

Results from real-time RT-PCR indicated that all three cell lines expressed hTERT mRNA (Figure 1C), and the TRAP assay confirmed that telomerase was active in these cell lines (Figure 1D). Based on the level of PPARγ mRNA and protein expression, and the presence of hTERT mRNA and telomerase activity, the MDA-MB-231 cell line was selected as an *in vitro *model for this study.

### Troglitazone reduces telomerase activity in a dose and time dependent manner

**Figure 2:**
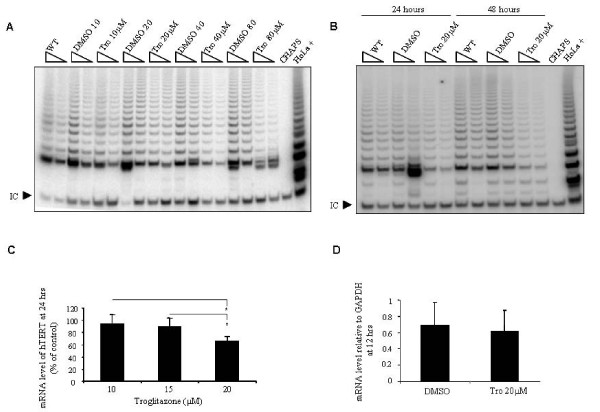
**Troglitazone suppresses the activity of telomerase in a dose and time dependent manner**. **A) **Protein lysates (500 and 250 ng respectively) from MDA-MB-231 cells with no treatment (WT), treated with various concentrations of troglitazone (Tro) or the equal volume of DMSO for 24 hours were examined for telomerase activity by the TRAP assay. **B) **MDA-MB-231 cells were treated with 20 μM of troglitazone (Tro 20 μM) and the equal volume of DMSO for 24 and 48 hours. Protein lysate (500 and 250 ng respectively) from non-treated cells (WT), Tro 20 μM, and DMSO was used for the TRAP assay. **A **and **B) **I.C., the internal PCR amplification control. CHAPS, lysis buffer only as a negative control. HeLa, 500 cell equivalent lysate as a positive control. **C) **Total RNA was collected from DMSO and troglitazone treated cells for 24 hours and the mRNA expression level was measured by real-time RT-PCR. Data were normalized against the DMSO treated group (control) in the respective treatment condition. **D) **Total RNA from MDA-MB-231 cells treated with DMSO and 20 μM of troglitazone (Tro 20 μM) for 12 hours was extracted and the mRNA expression of hTERT was analyzed by real-time RT-PCR. Values express the relative quantity of hTERT to GAPDH. The final percentage of DMSO did not exceed 0.1% v/v. **C **and **D) **results shown are expressed as mean ± SD and are representative of at least three independent experiments. * *p *< 0.05.

MDA-MB-231 cells were cultured in growth medium with increasing concentrations of troglitazone or equivalent volumes of DMSO for 24 hours. Visible inhibition of telomerase activity was observed at a troglitazone concentration of 20 μM, which increased at 40 μM and 80 μM of troglitazone (Figure 2A). Cells were treated with 20 μM troglitazone or an equivalent volume of DMSO for 24 or 48 hours. Cells were then harvested and telomerase activity was measured by the TRAP assay. The inhibition of telomerase activity using 20 μM of troglitazone at 48 hours was greater than at 24 hours. These results suggest that telomerase inhibition by troglitazone is both dose and time dependent, and that treatment of 20 μM of troglitazone for 24 hours was minimally sufficient for telomerase inhibition (Figure 2B).

### Troglitazone suppresses hTERT transcription

To test if lower concentrations of troglitazone reduce the level of hTERT mRNA, MDA-MB-231 cells were treated with concentrations of troglitazone less than 20 μM for 24 hours and the mRNA expression of hTERT was determined by real-time RT-PCR. Troglitazone exhibited a significant dose dependent reduction in the expression of hTERT mRNA compared to control at a concentration of 20 μM (*P *< 0.05) (Figure 2C). This result suggests that troglitazone suppressed telomerase activity by reducing the level of hTERT mRNA. However, cells treated for 12 hours with 20 μM troglitazone did not show significant changes in hTERT mRNA compared to controls (Figure 2D). Our results indicate that the minimum concentration and time to observe the effect of troglitazone on hTERT transcription and telomerase activity is 20 μM for 24 hours.

### Reduction in telomerase activity is independent of the transcriptional role of PPARγ

**Figure 3:**
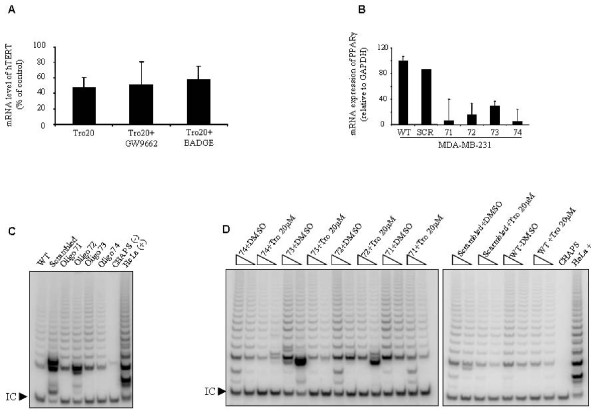
**The suppressive role of troglitazone on telomerase is independent from PPARγ**. **A) **MDA-MB-231 cells were exposed to either 10 μM GW9662 or 100 μM BADGE for 24 hours. Cells then were treated with 20 μM troglitazone in the presence of GW9662 or BADGE for another 24 hours. At the end of the incubation time, the expression level of hTERT was determined by real time RT-PCR. Values are expressed as the percentage of vehicle-treated controls (DMSO) in the respective treatment condition. Results shown are as mean ± SD and are representative of three independent experiments. **B) **Utilizing shRNA interference, the expression of PPARγ was inhibited with four different shRNA oligos (71, 72, 73, and 74) in the MDA-MB-231 cell line. To assess the specificity of shRNA oligos against PPARγ , MDA-MB-231 cells were transfected with scrambled oligo as a control. The mRNA level of PPARγ was examined by real-time RT-PCR. WT, non-infected cells; SCR, scrambled oligo; 71-74 shRNA oligos. **C) **The TRAP assay was used to examine the activity of telomerase in MDA-MB-231 cells in the absence of PPARγ (Oligo 71, 72, 73, 74) compared to non-infected MDA-MB-231 cells (WT) and cells transfected with a scrambled oligo which acted as non-silencing control shRNA sequence (SCR). (500 and 250 ng of cell lysate, respectively. **D) **MDA-MB-231 cells carrying silenced PPARγ by shRNA were treated with 20 μM troglitazone or the equal volume of DMSO for 24 hours and telomerase activity was measured using the TRAP assay (500 and 250 ng of cell lysate, respectively). **C **and **D) **I.C., the internal PCR amplification control. CHAPS, lysis buffer only as a negative control. HeLa, 500 cell equivalent lysate as a positive control. Result shown is representative of two independent experiments.

To assess the involvement of PPARγ in the reduction of telomerase activity, MDA-MB-231 cells were exposed to two different PPARγ antagonists, 10 μM GW9662 [33] and 100 μM of BADGE [34], for 24 hours prior to troglitazone treatment. Results from real-time RT-PCR showed that addition of either GW9662 or BADGE did not abrogate the suppressive effect of troglitazone on hTERT gene expression (Figure 3A).

Furthermore, we knocked down the expression of PPARγ using shRNA interference using lentiviral infection. Results from real-time RT-PCR showed that the expression of PPARγ mRNA in knocked-down cells was significantly decreased compared to wild type and scrambled shRNA infected cells (Figure 3B). With PPARγ expression significantly decreased, we examined the telomerase activity in the MDA-MB-231 cells and found that there were no significant changes in telomerase activity in knocked-down cells compared to wild type MDA-MB-231 cells (Figure 3C).

To examine the effect of troglitazone on telomerase activity in the absence of PPARγ, wild type and PPARγ knocked-down MDA-MB-231 cells were treated with 20 μM troglitazone for 24 hours. Our data showed that troglitazone was able to suppress telomerase activity in the absence of PPARγ transcription (Figure 3D).

### The cell toxicity effect of troglitazone

**Figure 4:**
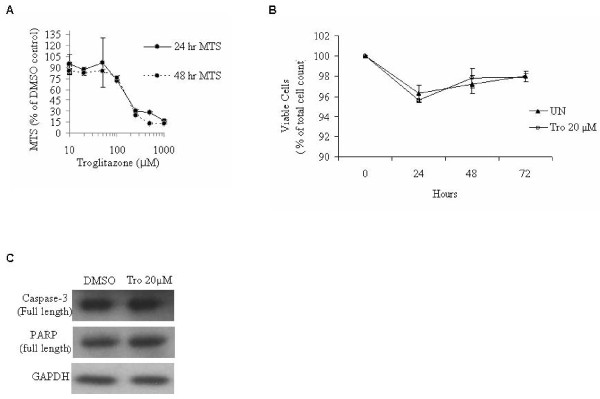
**Troglitazone does not cause cell toxicity and does not induce apoptosis in MDA-MB-231 cells**. **A) **MDA-MB-231 cells were treated with various concentrations of troglitazone (0, 10, 20, 50, 100, 250, 500, 1000 μM) for 24 or 48 hours and cell toxicity was determined by the MTS assay. All data were normalized against the DMSO treated control group in the respective treatment. **B) **Cells were exposed to 20 μM troglitazone for 3 days and cell viability was measured using automated Vi-CELL. Values expressed as percent survival of DMSO treated controls. Data were collected from three experiments performed in triplicate. WT, DMSO; T20, 20 μM troglitazone. **C) **Equal amounts of MDA-MB-231 cell lysate from DMSO and treated cells with 20 μM troglitazone (Tro 20 μM) for 24 hours were subjected to western blot analysis to determine the protein levels of caspase-3 and PARP. No differences were found between control and troglitazone treated cells. The western blots shown are representative of three separate experiments.

To determine the non-toxic concentration of troglitazone for cell treatment in our study, we measured the IC_50 _for troglitazone using the CellTiter96 non-radioactive proliferation assay kit. This assay measures the activity of dehydrogenase found in metabolically active cells. Dehydrogenase, a mitochondrial enzyme, reduces MTS (3-(4,5-dimethylthiazol-2-yl)-5-(3-carboxymethoxyphenyl)-2-(4-sulfophenyl)-2H-tetrazolium, inner salt) chemically into formazan [35]. Since the production of formazan is proportional to the activation of this enzyme in viable cells, the intensity of the produced color is an indicator of cell viability. The MTS result showed that the IC_50 _of troglitazone after 24 and 48 hours exposure was 190 μM (Figure 4A). Therefore, 20 μM of troglitazone for 24 hours is not toxic to MDA-MB-231 cells.

### Troglitazone does not induce apoptosis

It has been shown that PPARγ ligands induce programmed cell death (apoptosis) (for review see [36]). First, we examined the effect of troglitazone on cell viability using the trypan blue dye exclusion method. Cells treated with troglitazone showed a slight reduction in cell viability compared to controls (Figure 4B), however, this reduction was not statistically significant. We also studied the effect of troglitazone on caspase-3, the main apoptosis regulator. We found that the protein level of caspase-3 remained unchanged in response to troglitazone treatment (Figure 4C). Furthermore, we examined the protein levels of poly (ADP-ribose) polymerase (PARP), a protein which undergoes caspase-3 mediated cleavage during apoptosis and produces an 89 kDa fragment. In agreement with the caspase-3 results, we did not observe a reduction of PARP protein levels in troglitazone treated cells compared to DMSO treated cells (Figure 4C). These results confirm that troglitazone at 20 μM for 24 hours does not induce apoptosis and is not toxic to the cells.

### Troglitazone does not induce differentiation of MDA-MB-231 within 24 hours

**Figure 5:**
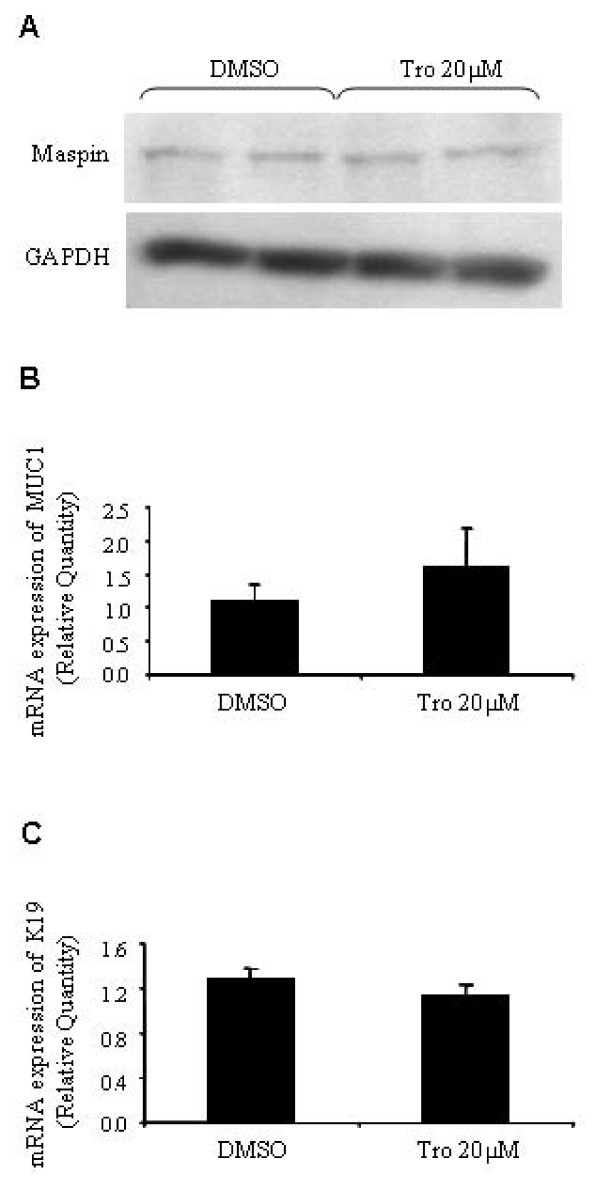
**Troglitazone does not induce cell differentiation**. MDA-MB-231 cells were treated with 20 μM of troglitazone (Tro 20 μM) or the equal volume of DMSO for 24 hours. **A) **Representative western blot showing the effect of 20 μM of troglitazone on the protein level of maspin. Western blots of total protein were probed with anti-maspin antibodies and then reprobed with anti-GAPDH to confirm equal loading. **B **and **C) **Total RNA from treated and control cells was used to examine the expression level of **B) **Mucin-1 (Muc-1) and **C) **Keratin 19 (K19) by real-time RT-PCR. Values express the relative quantity of the genes to the level of mRNA expression of GAPDH. Data shown is representative of three separate experiments.

Accumulating evidence indicates that PPARγ promotes cell differentiation following activation by its ligand [18-22] and since telomerase is not active in differentiated cells [37,38], we wondered if the inhibition of telomerase by troglitazone is the result of cell differentiation. MDA-MB-231 cells were treated with 20 μM of troglitazone for 24 hours and the expression of maspin was examined as a marker for differentiated breast epithelial cells [39]. As shown in Figure 5A, troglitazone did not increase the protein level of maspin, indicating that treated cells do not exhibit significant differentiation at 24 hours post-treatment. We also measured the expression of two genes associated with breast malignancy, Keratin 19 (K19) and mucin-1 (Muc-1) as previously described [19]. Real-time RT-PCR showed the expression of these two genes was unchanged in troglitazone treated cells (Figure 5B and 5C).

### The expression of hTERT is not correlated with the expression of PPARγ in clinical samples

**Figure 6:**
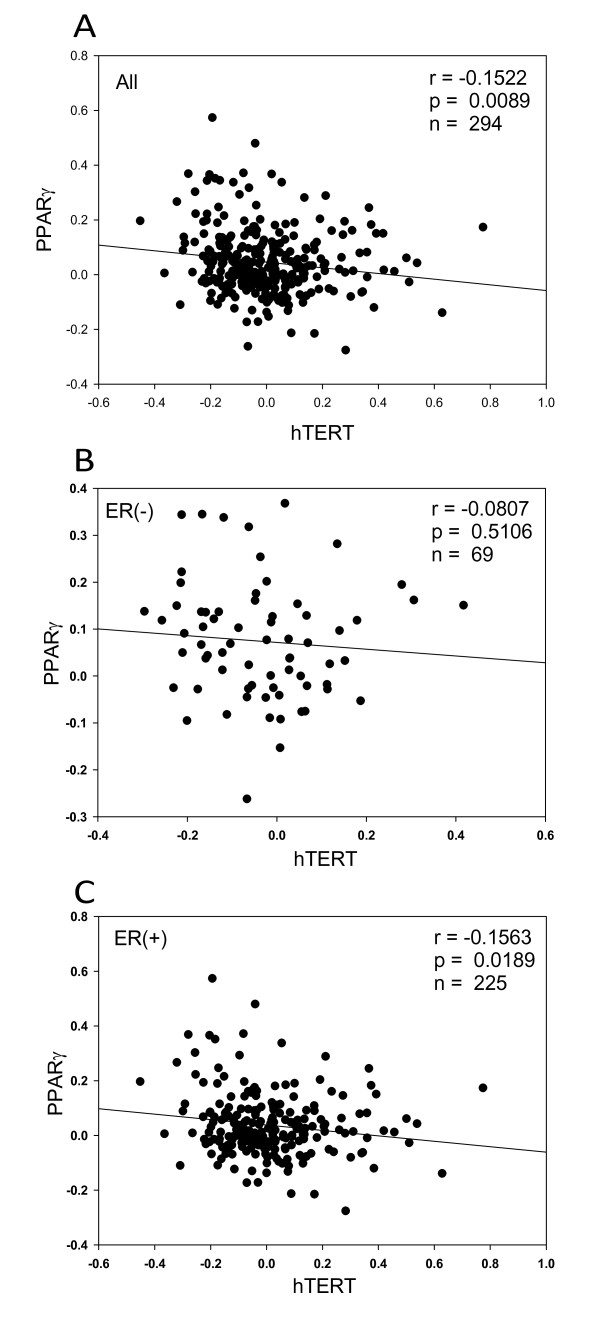
**The expression of hTERT is independent from PPARγ**. Genome-wide gene expression microarray data for 294 patients with early breast cancers from the NKI published dataset was used to measure the correlation between hTERT and PPARγ expression in A) all 294 tumor samples with early breast cancers (r = -0.1522)(p = 0.0089), B) 69 estrogen receptor negative tumors (r = -0.0807)(p = 0.5), and C) 225 estrogen receptor-positive tumors (r = -0.1563)(p = 0.0189).

Published NKI data from 294 young patients with primary invasive breast cancers was used to compare the expression of PPARγ and hTERT [32,40]. We found that the expression of PPARγ and hTERT in this set of samples was 44% and 62% respectively (data not shown). Our results show that there is no correlation between the level of PPARγ expression and hTERT expression (r = -0.152) in these samples (Figure 6A). Since MDA-MB-231 cells are estrogen receptor negative [41], we compared the expression of PPARγ and hTERT genes in estrogen receptor-negative (N = 69) and estrogen receptor-positive (N = 225) tumor samples and no correlation was observed between the two genes in either estrogen receptor-positive (r = -0.156) (Figure 6B) or estrogen receptor-negative (r = -0.08) (Figure 6C) tumors.

## Discussion

Breast cancer, the most common malignancy in women, is a heterogeneous disease currently treated with surgery, chemotherapy and radiation. All of these methods treat the cancer, but affect healthy tissue as well. There is a desperate need for new therapies to treat breast cancer as many cases fail to respond to current chemotherapeutic agents. Targeted cancer therapies focus on specific molecules that influence the growth and spread of cancer cells. ER was the first protein that was successfully targeted. More recently trastuzumab (Herceptin) has been introduced for the treatment of women with HER2 positive breast cancers. Inhibition of telomerase has been found to limit the growth of human cancer cells (reviewed in [4]). Telomerase is active in 90% of breast cancers [5-7], which make it a promising potential target for breast cancer treatment. There is some evidence indicating that PPARγ may play a critical role in this process [27-29,42]. The objective of this study was to investigate the effect of troglitazone on telomerase as a potential novel therapeutic approach that could be used to treat breast cancer. To our knowledge, this is the first time that the effect of troglitazone on telomerase activity in breast cancer has been studied.

We studied three human breast cancer cell lines; MDA-MB-231, MCF-7, and T47D. MDA-MB-231 is estrogen receptor negative [41], EGF responsive and IGF-I non-responsive. MCF7 is estrogen receptor positive [41], EGF non-responsive and IGF-I responsive and T47D is estrogen receptor positive, EGF responsive and IGF-I non-responsive [43]. Real-time RT-PCR data showed the expression of PPARγ mRNA was higher in MDA-MB-231 cells compared to MCF-7 and T47D. Western blot analysis confirmed that all three cell lines express detectable amounts of PPARγ protein in agreement with the results from other investigators [44,45]. We also validated the expression of hTERT and telomerase activity in these cell lines. Real-time RT-PCR showed that all three cell lines express hTERT mRNA. This data has been confirmed by the TRAP assay, indicating that telomerase is active in all three cell lines.

It has been shown that 17β-estradiol (E2) up-regulates telomerase activity in ER-positive breast cancer models both *in vivo *and *in vitro *[46-48]. Using ER-positive MCF-7 breast cancer cells, it has been found that the activation of telomerase is accompanied by up-regulation of hTERT mRNA [47,48]. Evidence indicates that estrogen activates hTERT directly by binding to estrogen response elements (EREs) found in the promoter of hTERT and indirectly through activation of c-Myc expression in MCF-7 cells [47,48]. However, a similar result in ER-negative cells was not observed, suggesting a regulatory role for estrogen that is limited to ER-positive cells [47,48].

To study the effect of PPARγ on telomerase activity, the MDA-MB-231 cell line was chosen as an estrogen receptor negative model because of the presence of active telomerase and high expression of PPARγ protein. Using the TRAP assay, we showed that troglitazone suppresses telomerase activity specifically. The presence of an internal control in the TRAP assay indicated that the suppression of telomerase activity was not due to inhibition of PCR amplification.

We also found that hTERT mRNA levels were significantly reduced by troglitazone. Our result showed that 20 μM of troglitazone was the minimum concentration able to suppress telomerase activity after 24 hours. We also found that troglitazone at this concentration was not toxic to the cells indicating that the inhibition of telomerase by troglitazone is not a consequence of cell toxicity.

There is accumulating published data indicating that troglitazone can act independently from PPARγ [17,49]). In addition, it has been shown recently that thiazolidine derivatives unable to activate PPARγ have an antiproliferative effect in both hormone-dependent and hormone independent breast cancer cell lines [50].To study the role of PPARγ in modulating the expression of hTERT and telomerase activity, we studied the effects that two structurally different PPARγ antagonists, BADGE and GW9662 have upon troglitazone inhibition of hTERT expression. We found that neither BADGE nor GW9662 were able to prevent the suppressive effect of troglitazone on hTERT expression. This finding was confirmed using shRNA silencing of *PPARγ*. We showed that troglitazone suppresses telomerase even in the absence of PPARγ mRNA expression. This result indicates that the effect of troglitazone on telomerase activity is independent from PPARγ. There is some evidence showing that activated PPARγ abolishes telomerase activity [27-29,42]. It is noteworthy that none of these groups used MDA-MB-231 cells as their models. Moreover, none of them used troglitazone as the ligand for PPARγ. Recently, using pancreatic cancer cell lines, Kondoh *et al. *showed that 15-deoxy-Δ^12,14 ^prostaglandin J_2 _(15d-PGJ_2_), a natural ligand for PPARγ, suppresses the expression of hTERT by blocking ER functions [51]. However, this group did not demonstrate whether this suppression is mediated through PPARγ activation. Although the underlying mechanism for these differences remains to be discovered, it may reflect differences in experimental models and approaches such as cell type, particular ligand, duration of treatment, and dosage.

To examine whether the suppression of telomerase is the result of apoptosis induction, we investigated the effect of troglitazone on cell viability and the protein levels of caspase-3 and PARP as apoptosis markers. Although it has been shown that troglitazone induces apoptosis in different cancer cell lines by different mechanisms [52-55], our result showed that troglitazone does not induce apoptosis in MDA-MB-231 cells at this concentration. It is noteworthy that these groups used a higher concentration of troglitazone. Published data from other groups indicates that a low concentration of troglitazone cannot promote apoptosis. Elstner *et al. *showed that 10 μM troglitazone does not induce apoptosis in the MCF-7 cell line after 4 days incubation [56]. In agreement with this study, Ohta *et al. *demonstrated that although 10 μM troglitazone can induce DNA fragmentation in BHP18-21, a thyroid papillary carcinoma cell line, it was not able to change the expression level of *bcl-2 *and *bax *genes. Interestingly, 10 μM troglitazone induced DNA fragmentation when BHP18-21 cells were cultured in 0.1% v/v fetal calf serum (FCS). Conversely, under 10% v/v FCS conditions, more than 100 μM troglitazone was required to induce DNA fragmentation [57]. In agreement with this result, we found that 20 μM troglitazone induces apoptosis in our cell culture model in 0.1% v/v FBS media after 24 hours incubation (data not shown). The differences in troglitazone concentration, culture conditions, and cell types may explain these differences. Importantly, all these groups showed that regardless of the concentration of troglitazone used, the induction of apoptosis was independent from PPARγ activity [52-57]. Since the promotion of apoptosis was not observed in our cell model, it suggests that the suppression of hTERT and its telomerase activity by troglitazone is not due to apoptosis activation.

Although PPARγ can promote cell differentiation [18-22], this effect has not been observed in all studies [56]. Since differentiated cells do not generally possess detectable telomerase activity [37,38], we investigated if telomerase suppression in our study was the result of cell differentiation. MDA-MB-231 cells were exposed to various concentrations of troglitazone for different time periods. We examined the expression of three different markers associated with breast cancer. It has been shown that the mRNA expression of maspin is decreased in malignant breast cells compared to normal breast epithelial cells [39]. In contrast, K19 and Muc-1 are associated with more malignant breast epithelial cells [19]. Our results showed that troglitazone was not able to change the expression of these genes within 24 hours, indicating that treated cells had not become differentiated during this time. Furthermore, by increasing the concentration of troglitazone, we did not observe any signs of cell differentiation but we observed cell toxicity and apoptosis. This result suggests that troglitazone at low concentrations inhibits telomerase activity independently from cell differentiation, and is an effect that cannot be ascribed to cell toxicity or apoptosis.

There is a significant correlation between hTERT mRNA and telomerase activity in human breast carcinoma tissue [58]. To compare our findings with what has been observed in clinical samples, we analyzed the expression of PPARγ and hTERT from genome-wide gene expression microarray data for 294 patients [32,40]. We found no correlation between these two genes (r = -0.152). Furthermore, since the MDA-MB-231 cell line is an estrogen receptor negative cell line, we compared the expression of hTERT and PPARγ in estrogen receptor (ER) negative patients. No correlation was found between the expression of hTERT and PPARγ (r = -0.08) in this group of patients. In agreement with our *in vitro *model, this result suggests that telomerase inhibition by PPARγ ligands is independent from PPARγ transcriptional activity and is effected by an unknown mechanism.

## Conclusions

To our knowledge this is the first time that the effect of troglitazone on telomerase activity has been studied in human breast cancer. We showed that the expression of hTERT and PPARγ are two independent events and troglitazone reduces the activity of telomerase by recruiting other pathway(s) rather than PPARγ activity. Our study shows although the mechanism underlying this suppression remains unclear, and may be indirect, troglitazone can be considered as an anti-telomerase agent in estrogen-receptor negative breast cancer cells. In addition, based on data from our studies as well as others, we suggest that the role of troglitazone, and probably the other members of TZD family, should be revisited beyond their original role merely as PPARγ ligands.

## Abbreviations

GAPDH: glyceraldehyde-3-phosphate dehydrogenase; hTERT: human telomerase reverse transcriptase; hTR: human telomerase RNA; NKI: Netherlands Cancer Institute; PARP: poly (ADP-Ribose) polymerase; PPARγ: peroxisome proliferator activated receptor gamma; RXR: retinoid X receptor; TRAP: telomeric repeat amplification protocol; TZD: thiazolidinedione

## Competing interests

The authors declare that they have no competing interests.

## Authors' contributions

FRK conceived, designed and carried out the experiments and drafted the manuscript. MT assisted with experimental design, performed the TRAP assays and assisted with the manuscript preparation. JN performed the real time RT-PCR analyses. DYW performed the microarray dataset extraction and statistical analyses. LH assisted with experimental design and manuscript preparation. SD obtained funding for the study, supervised the project, and assisted with experimental design and manuscript preparation. All authors read and approved the final manuscript.

## Authors' information

SD acknowledges the support of the Canadian Breast Cancer Research Alliance.

LH acknowledges the support of the National Cancer Institute of Canada (15072) with funds from the Canadian Cancer Society.

MT acknowledges the support of the Canadian Breast Cancer Foundation - Ontario Chapter.

This research was funded in part by the Ontario Ministry of Health and Long Term Care. The views expressed do not necessarily reflect those of the OMOHLTC.

## Pre-publication history

The pre-publication history for this paper can be accessed here:

http://www.biomedcentral.com/1471-2407/10/390/prepub
